# Femtosecond laser modification of an array of vertically aligned carbon nanotubes intercalated with Fe phase nanoparticles

**DOI:** 10.1186/1556-276X-8-375

**Published:** 2013-09-03

**Authors:** Vladimir Labunov, Alena Prudnikava, Serguei Bushuk, Serguei Filatov, Boris Shulitski, Beng Kang Tay, Yury Shaman, Alexander Basaev

**Affiliations:** 1 Laboratory of Integrated Micro- and Nanosystems, Belarusian State University of Informatics and Radioelectronics, P. Brovka St. 6, Minsk 220013, Republic of Belarus; 2 Laboratory of Optical Diagnostics, B.I. Stepanov Institute of Physics of the National Academy of Sciences of Belarus, Minsk 220072, Republic of Belarus; 3 Laboratory of Hydrogen Energy, Institute of Heat and Mass Transfer of the National Academy of Sciences of Belarus, Minsk 220072, Belarus; 4 School of Electrical and Electronic Engineering, Nanyang Technological University, Singapore 639798, Singapore; 5 Scientific-Manufacturing Complex Technological Centre MIET, K-498, Moscow 103498, Russia

**Keywords:** Femtosecond laser ablation, Carbon nanotube array, Iron phase nanosphere, Chemical vapor deposition, 61.48.De, 06.60.Jn, 42.62.Cf

## Abstract

Femtosecond lasers (FSL) are playing an increasingly important role in materials research, characterization, and modification. Due to an extremely short pulse width, interactions of FSL irradiation with solid surfaces attract special interest, and a number of unusual phenomena resulted in the formation of new materials are expected. Here, we report on a new nanostructure observed after the interaction of FSL irradiation with arrays of vertically aligned carbon nanotubes (CNTs) intercalated with iron phase catalyst nanoparticles. It was revealed that the FSL laser ablation transforms the topmost layer of CNT array into iron phase nanospheres (40 to 680 nm in diameter) located at the tip of the CNT bundles of conical shape. Besides, the smaller nanospheres (10 to 30 nm in diameter) are found to be beaded at the sides of these bundles. Some of the larger nanospheres are encapsulated into carbon shells, which sometime are found to contain CNTs. The mechanism of creation of such nanostructures is proposed.

## Background

Ultrafast lasers are playing an increasingly significant role in materials research, characterization, and surface morphology modification due to a number of unexpected phenomena and formation of new structures. For the past 10 years, being the leading material in semiconductor and photonic industries, silicon has attracted majority of interest and the modification of its surface morphology in different environments using the femtosecond laser (FSL) irradiation has been intensively studied [1-9].

The initial discovery was made when a polished silicon surface was transformed into a forest of quasi-ordered micrometer-sized conical structures upon exposure to several hundred FSL pulses in an atmosphere containing sulfur hexafluoride (SF_6_) [10,11]. These conical structures could trap a large quantity of sulfur doping the semiconductor at a concentration that was well above the solubility limit. The confluence of these chemical and structural changes has yielded a unique new material with novel optical properties that have never been observed. This material absorbed about twice as much of visible light as compared to normal silicon and had the ability to detect infrared light that is invisible to the current generation of silicon detectors; also, it possessed significant advantages in detection, imaging, and power generation applications [3,9,12-14]. Lately, various morphology and property changes were reported that have resulted from the FSL irradiation with different varieties of ambient, including various gaseous, as well as vacuum, liquid, water, and air [2,5,6,15-17].

Over the past two decades, carbon nanotubes (CNTs) have attracted a lot of attention due to their exceptional properties [18,19], and as it is expected, potentially, they can replace silicon in the emerging nanoelectronics and nanophotonics.

Hence, investigating the interaction of FSL irradiation with CNTs would represent a great interest. The first result that is useful for our investigation was obtained while studying the light interaction with fluffy arrays of single and multiwall CNTs containing metal (Fe) catalyst nanoparticles using a photoflash [20-24]. Photoacoustics and ignition have been observed in these arrays. The mechanism of ignition was attributed to the light absorption by CNT arrays due to the black body effect that generated rapid increase in temperature. As a result, a chain oxidation reaction of CNTs and metal nanoparticles was initiated which caused ignition; as a result of which, nanoparticles containing Fe_2_O_3_ and Fe_3_O_4_[24] or C, O, Si, and Fe [23,24] were produced. The most important result of this investigation was that the metal nanoparticles are playing significant role in the deposited energy absorption.

A number of investigations were performed with the laser irradiation of arrays of dense vertically aligned CNTs which have been pursuing the aim of pattering the arrays in order to obtain the configurations of some devices. This process is known as laser pruning [25], burning [26], or laser machining [27]. The lowering of the nanotube density and formation of nanotube junctions and nanoparticles via laser surface treatment were well reported [25-27]. To our best knowledge, only in few works, the femtosecond laser pulses were used for CNT treatment, for example [27,28], while in the rest continuous irradiation of the gas or solid state lasers was utilized [25,26,29,30]. What is important is that in all aforementioned studies, CNT arrays were synthesized by different chemical vapor deposition (CVD) methods, either thermal, hot filament, or plasma enhanced, but in all of them, the localized on the substrate catalysts (Fe or Al/Fe) were used. As a result of this technology utilization, the CNT arrays did not contain metal catalyst nanoparticles. This situation determines the interaction process itself and the obtained products of interaction and could be considered as the simplest case of the laser irradiation interaction with the CNT arrays.

In the present work, we investigate for the first time the interaction of femtosecond laser irradiation with the arrays of vertically aligned carbon nanotubes intercalated with the ferromagnetic (Fe phase) nanoparticles. The presence of metal nanoparticles in CNT array, as it was shown in [20-23], plays the important role in the energy absorption by the array. The importance of the present investigation is defined by the possible applications of the obtained results. The arrays of CNTs with the intercalated ferromagnetic nanoparticles, so called magnetically functionalized CNTs (MFCNTs) [31,32], may be considered as an ideal medium for different magnetic applications. They can be used as sensors, sensitive elements of magnetometers, magnetic filters, ferrofluids, xerography, magneto-resonance imaging, magnetic hypothermia, and biomedical applications. The superior application of oriented MFCNT arrays can be in a sphere of magnetic write/read heads and high-density data storage devices [33-36]. The FSL irradiation may become an instrument for the machining of the mentioned devices based on the arrays of MFCNTs.

In particular, in the present work, we investigate the surface morphology modification of the vertically aligned MFCNTs upon FSL irradiation and properties of the products obtained after irradiation and develop the mechanism of the interaction of FSL with such complicated media as the arrays of MFCNTs.

## Methods

CNT arrays were synthesized on Si substrates by the floating catalyst CVD via a high-temperature pyrolysis of the xylene/ferrocene solution injected into the reaction zone of quartz reactor. In our particular case, the concentration of ferrocene in the solution was 10%; the temperature in the reaction zone was 875°C, and the process duration was 30 s. Obtained as a result of ferrocene decomposition, Fe phase nanoparticles serve as catalyst for CNTs growth. During the growth process, these nanoparticles are intercalating into CNT arrays and are considered as fillers of CNTs.

The morphology of the CNT arrays before and after the FSL irradiation was investigated by scanning electron microscopy (SEM) (Hitachi S-4800 FE-SEM, Chiyoda-ku, Japan). For Raman measurements, Renishaw micro-Raman Spectrometer (Series1000, Renishaw, Wotton-under-Edge, UK) with laser beam of 1.5 mW incident power and 514 nm wavelength was used. The structure of CNTs was characterized by transmission electron microscopy (TEM, JEM 100-CX, JEOL) and a high-resolution TEM (JEM-2010, JEOL Ltd., Akishima-shi, Japan). For X-ray diffraction analysis (XRD), DRON-3 diffractometer (Bourevestnik, Inc., Maloochtinskiy, Russia) was used; the local configurations of iron ions of CNTs fillers were examined with Mössbauer spectroscopy (spectrometer MS2000 with Fe/Rh source, 40 mCu). Elemental analysis was made by energy-dispersive X-ray spectroscopy (EDX) (SUPRA-55WDS with the EDX prefix, Carl Zeiss, Inc., Oberkochen, Germany).

The FSL irradiation was performed in ambient atmosphere with laser scanning microscope (Zeiss LSM 510 NLO based on Zeiss Aviovert 200 M inverted research microscope). This setup is equipped with femtosecond titanium-sapphire laser (Spectra-Physics Tsunami, Santa Clara, CA, USA) delivering 100 fs pulses at a wavelength of 790 nm with 82 MHz repetition rate. The energy of a single pulse was 15 nJ. The laser beam was then focused by Zeiss Plan-Neofluar 40x/0.75 objective and formed a spot with 1.2 μm in diameter on the sample surface. The beam was attenuated with an acoustic-optical filter to the energy level of 6.25nJ per pulse at the focal plane of the microscope objective. The investigated samples were placed onto the stage of the microscope without cover glass. CNT array treatment was achieved by scanning line-by-line at 512 lines per scan resolution. The scan speed was about 145 mm/s. The dimension of the scan area could be varied from 230 × 230 μm to 30 × 30 μm. Zoom factor of the microscope was chosen equal or greater to the required Nyquist criterion to ensure the focal spot overlaps between neighboring lines. Three-dimensional scanning is achieved with a built-in *Z*-axis drive. The step of *Z*-axis was chosen to be 1 μm, again to ensure the spatial overlapping of the focal spot between neighboring planes.

## Results

**Figure 1:**
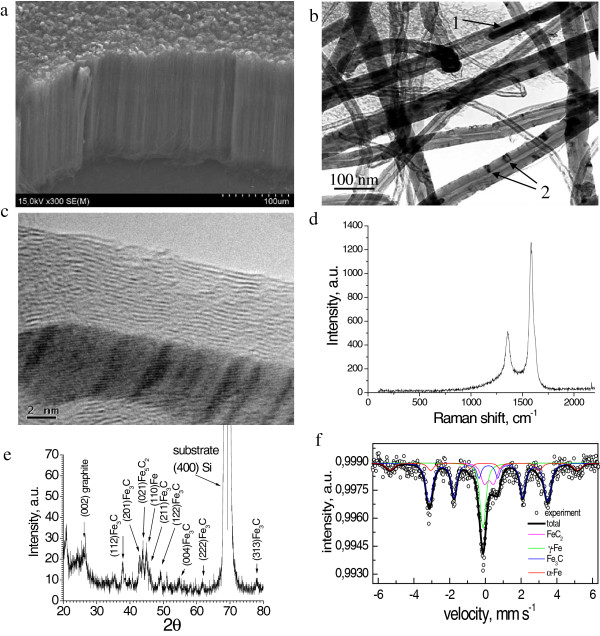
**Characteristics of the obtained CNT array grown on Si substrate. (a)** Morphology (SEM). **(b)** TEM image of CNTs with the filler in the CNTs channels (1) and walls (2). **(c)** HRTEM image of a multiwall CNT with the filler in its channel. **(d)** Raman spectrum. **(e)** XRD pattern. **(f)** Mössbauer spectrum.

The characteristic morphology and composition of the obtained CNT array as well as the CNT structure are depicted in Figure 1a,b,c,d,e,f. Figure 1a shows the SEM image of the synthesized dense vertically aligned CNT array. Figure 1b,c shows the TEM images of the synthesized CNTs which are found to be multiwall, with outer diameters of 12 to 70 nm. From Figure 1b, it is seen that some CNTs are filled with nanoparticles (1) in the channels of CNTs and (2) in between their walls. Figure 1d corresponds to the Raman spectrum collected from the sample which contains *D* peak (approximately 1,358 cm^−1^) arising from the structural disorder and *G* peak (approximately 1,584 cm^−1^) common to all sp^2^ carbon forms. The ratio of intensities *I*_G_/*I*_D_ = 2.47 testifies that CNTs are well crystallized and have low defect concentration. The XRD pattern in Figure 1e shows that the CNT array contains graphite (002) with a rhombohedral structure [37] (ICDD card no. 75–2078, PCPDFWIN), which is a characteristic of CNTs. Besides, the XRD pattern exhibits a series of peaks corresponding to Fe phase (including carbides): Fe_3_C and Fe_5_C_2_. Analysis of the XRD result reveals that carbide Fe_3_C with an orthorhombic structure (space group Pbnm) dominates over the other phases of nanocomposite (approximately 90%) [32,38]. The Mössbauer spectrum collected in transmission geometry at room temperature is shown in Figure 1f, and the hyperfine parameters (subspectra) are summarized in Table 1. It has been specified that these states of iron are fcc γ-Fe, bcc α-Fe, and Fe_3_C. However, the spectrum does not reveal the state of Fe_5_C_2_ but instead the doublet of FeC_2_. This discrepancy can be attributed to the difference in sensitivity between the two methods.

**Table 1:** **Hyperfine parameters of the Mössbauer spectrum shown in Figure **1**f**

**Subspectrum**	***δ***	***ΔЕ***	***Н***_***eff***_	**Contribution**	**Phase**
	**(mm/s)**	**(mm/s)**	**(T)**		
Singlet С	−0.13	0	-	20	γ-Fe
Doublet D	0.20	0.52	-	13	FeC_2_
Sextet S_1_	0.17	0	20.6	54	Fe_3_C
Sextet S_2_	−0.06	−0.03	32.6	13	α-Fe

**Figure 2:**
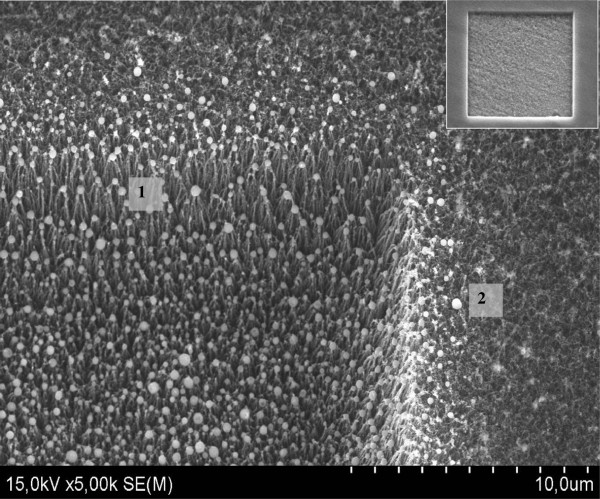
**Surface morphology of the FSL irradiated area of the CNT array (SEM).** (1) Nanospheres located at the tips of the CNT bundles. (2) Nanospheres located on top of CNT array (outside of the cavity). Inset: the entire 200 × 200 μm^2^ laser-processed surface.

A SEM image of the FSL irradiated area of CNT array is presented in Figure 2. The size of the irradiated zone is 200 × 200 μm^2^ (Figure 2, inset). It can be observed that upon the FSL irradiation, a square cavity of approximately 10 μm in depth was created. Nanoparticles of spherical shape were found at the bottom of the cavity located at the tips of conical shape CNT bundles. It is more prominent to observe these nanostructures at the walls of the cavity (indicated as ‘1’ in Figure 2). Also, some of these nanospheres (indicated as ‘2’ in Figure 2) are found to be sited slightly away from the irradiated area.

**Figure 3:**
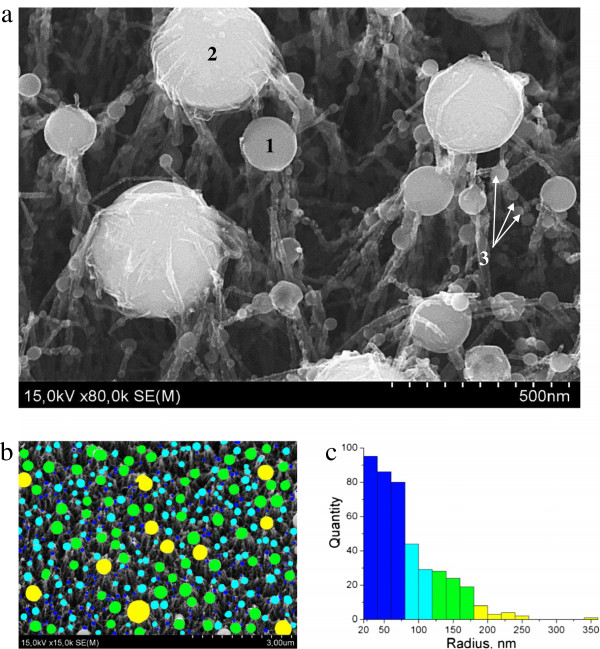
**SEM images of the nanospheres and their quantitative size distribution. (a)** An image of the nanospheres (SEM). (1) A nanosphere without a shell. (2) A nanosphere with the attached CNTs (might be covered with a shell), and (3) the nanospheres beading the CNT bundles. **(b)** Representative grouping of the nanospheres. **(c)** Corresponding size distribution.

In Figure 3a, the SEM image of the irradiated area is presented. It is seen that the nanospheres found at the tips of the CNT bundles (1,2) generally have a larger diameters, while those that are found to be beading the CNT bundles (3) have the smaller ones (approximately 10 to 30 nm). From Figure 3a, it is clearly seen that there are two types of larger nanospheres. Some of them are enveloped by the shells of a very complicated structure (2), whereas others do not have shells (2).

In Figure 3b,c, the quantitative analysis on the size distribution of the nanospheres of type (1, 2) is presented. It is seen that these nanospheres have a wide radius distribution (20 to 340 nm) with predominant radius in the range of 30 to 70 nm.

**Figure 4:**
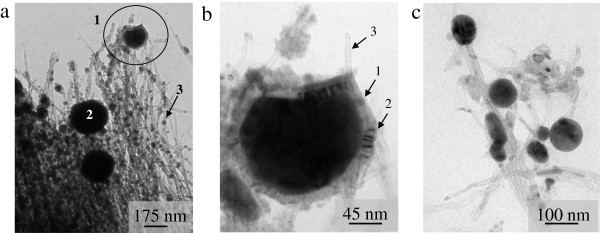
**TEM images of the nanospheres in contact with CNTs in the irradiated area. (a)** Nanospheres of larger diameter with (1) and without (2) shells found at the tip of the thinned CNTs, and (3) nanospheres of smaller diameter beading CNTs. **(b)** Enlarged view of the nanosphere encapsulated into the shell (1) containing some inclusions (2) and CNTs (3). **(c)** The nanospheres without shells.

The TEM images are presented in Figure 4a,b,c. In Figure 4a, it can be seen clearly that some of the nanospheres are encapsulated within a shell (1), while some are not (2). Besides, the diameters of CNTs attached to the nanospheres are found to be smaller (approximately 5 nm), as compared to CNTs before laser irradiation (Figure 1b). Smaller nanospheres can also be seen attaching to the outer walls of CNTs (3). Figure 4b represents big size nanospheres covered by the shell (1), which contains some inclusions (2) and a number of easily recognized CNTs (3), whereas in Figure 4c, the magnified image of the nonencapsulated nanosphere is shown.

**Figure 5:**
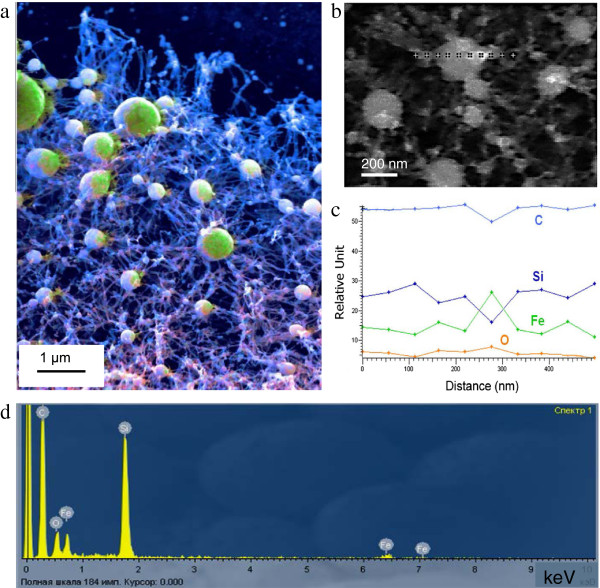
**EDX spectroscopy data on the composition of the FSL-irradiated CNT array on Si substrate. (a)** EDX image of the investigated area. **(b**, **c)** Element distribution along the diameter of the nanosphere. **(d)** EDX spectrum.

The EDX spectroscopy was employed in order to obtain a general overview of element distribution in the formed structure (Figure 5a,b,c,d). To have a better understanding within the nanostructure, partial of the CNT array was removed with a high-intensity FSL beam. The corresponding EDX image of the investigated area is shown in Figure 5a. In this figure, dark blue region corresponds to Si substrate, blue corresponds to CNTs, and green represents the nanospheres. Figure 5d shows the EDX spectrum demonstrating signals of Si, O, Fe, and C.

The in-depth quantitative analysis of the elemental composition within the nanosphere was obtained with a localized EDX analysis across its diameter with a 30-nm diameter electron beam spot. In Figure 5b, ten scanning spots across a 600-nm diameter nanosphere are depicted and in Figure 5c, the corresponding EDX analysis plot. It is shown that the composition near to the core of the nanosphere (between 160 and 380 nm of distance) has a higher content of Fe and O as compared to the outer layer of the nanosphere, where C and Si contents are higher. This fact testifies that the nanosphere composition is mainly Fe and O.

## Discussion

The removal of the topmost layer of the CNT array and the creation of a cavity upon the FSL irradiation are achieved by means of ablation. The ultrashort pulse ablation process includes the absorption of optical radiation by bound and free electrons of the material, energy transfer to the lattice, bond breaking, followed by evaporation of the material in a form of atoms or ions, and vapor expansion into an ambient gas. Usually, weak plasma is formed over the irradiated surface. The sputtered particles, upon losing energy, aggregate into clusters of different sizes, charges, and kinetic energies. These resulting clusters can be either carried away from the reaction zone or re-deposited back onto the target (substrate) surface. This process is known as laser machining; however, no adequate mechanism for the latter has been proposed.

**Figure 6:**
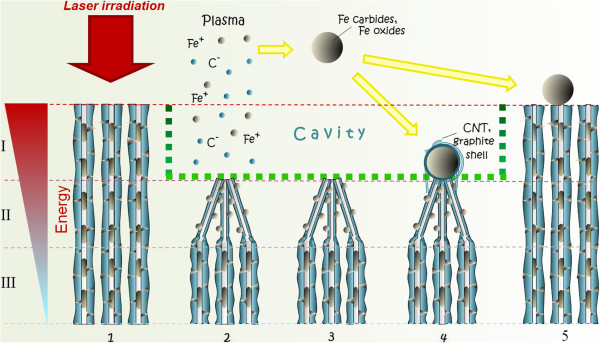
Schematic of the proposed mechanism of the interaction of the FSL irradiation with CNT arrays.

Here, we propose a basic scheme of formation of the cavity with the observed nanostructures as a result of the interaction of FSL irradiation with the arrays of the vertically aligned CNTs intercalated with Fe phase catalyst nanoparticles. Figure 6 shows the schematic of the proposed mechanism.

In our case, the CNT array represents the target for ablation that consists of two materials, i.e., graphitic CNT walls and various iron phase intercalated within the CNT channels and walls (Figure 6 (1)). Once the ablation threshold is reached, the topmost layer starts to ablate away, i.e., both CNTs and the Fe phase nanoparticles. The ablation of the two materials (C and Fe) occurs since the energy density even of a single pulse (0.48 J/cm^2^) exceeded both of the reported ablation thresholds of various carbonaceous materials (multiwall CNTs, 0.046 J/cm^2^[39]; single wall CNTs, 0.05 J/cm^2^[40,41]; graphite, 0.13 J/cm^2^[42]; graphene, 0.20 J/cm^2^[43]); and the ablation threshold of iron, 0.18 to 0.19 J/cm^2^[44,45]. The gradual ablation of the CNT array leads to the formation of the cavity of approximately 10 μm depth. This ablation process of the C-Fe target is rather complicated since two distinct materials are being subjected simultaneously to multiple ultrashort laser pulses during 3D scanning. It was found that the mechanism of solid ablation by the intense FSL irradiation is essentially the same [46]. Usually, at atmospheric pressure, the ablation process occurring near to the threshold is always initiated by the ultrafast melting (bonds breaking) of the material, which applies for iron. However, as it was shown by Jeschke's group [47], graphite has the unique property of exhibiting two distinct laser-induced structural instabilities. At high absorption energies regime (>3.3 eV/atom), nonequilibrium melting occurs that is followed by a fast evaporation. For low intensities, slightly above the damage threshold (>2.0 eV/atom), ablation occurs via removal of intact graphite sheets. Taking into account that the energy density of a single pulse equals to *F*_1_ = 0.48 J/cm^2^, we calculated the absorbed energy per atom *E*_0_ using the equation [48]:

(1) $$F_{1}=\frac{eE_{0}n_{a}d}{1-R-T},$$

where *e* is the Coulomb constant, *n*_a_ is the atomic density, *d* is the penetration depth of the light, *R* = 0.3 is the reflectivity, and *T* = 0 is the transmission of the material which were assumed to be as for graphite [48]. The penetration depth was calculated using the Drude formula *d* = *λ*/4π*k* with the wavelength of 790 nm and extinction coefficient *k* = 1.5 as for graphite [42]. It has been estimated that the atomic density of our CNT arrays is approximately *n*_a_ = 7.52 × 10^21^ atoms/cm^3^ which is lower than that of the graphite (*n*_a_ = 1.76 × 10^23^ atoms/cm^3^). The calculated value of the absorbed energy per atom even for a single pulse, *E*_0_ = 66.95 eV/atom, is much higher than those mentioned in [47] which implies that CNTs in these conditions are burnt instantly.

As a result of C and Fe ablation, localized weak plasma is formed over the irradiated surface (Figure 6 (2)). Both substances exist in their plasma state for a short period of time, while Fe undergoes a rapid condensation and aggregates into spherical clusters (thermodynamically the most favorable shape).

The energy density of the FSL beam, as it is shown in Figure 6, reduces along the depth of CNT array in the process of their interaction. At a certain depth (labeled as ‘II’), the energy is not sufficient for the CNT covalent bonds breaking and complete CNTs ablation. Only some of the external walls of the multiwall CNTs are ablated, and this leads to the thinning of the CNTs. The bundling of thinned CNTs into the cones can mainly be caused by the Van der Waals force or/and the magnetic interaction of Fe phase nanoparticles. The Fe phase inclusions located in between the CNT walls most probably have not undergone the complete evaporation but have been subject to a quick melting and resolidification; this led to the formation of smaller nanospheres beading the conical shape of CNT bundles (Figure 6 (3)).

Noteworthy that the Fe phase transformations occur in the presence of carbon atoms and though conditions are quite similar to the floating CVD method, one can suppose that Fe particles can serve as a catalyst for the formation, during the cooling process, of graphitic architectures (shells), covering the iron phase nanospheres. The shells sometime contain CNTs, (Figure 4a,b, Figure 6 (4)). Besides, it was reported that multiwall CNTs and onions had been obtained from graphite in vacuum at 7.5 J/cm^2^ FSL fluence with the estimated growth time of 1 to 2 ns [49]. Similar to the case of CNTs synthesis process, due to the stochastic process, not all of the catalyst particles facilitate the growth of graphitic shells.

The iron phase nanospheres (with and without shells), after their creation during the first FSL scans, freeze and deposit on the surface of the irradiated area, while some of them are sited slightly away (Figure 2).

During 3D scanning, the Fe-phase nanoparticles that are sited nearer to the tip of the CNTs (labeled as ‘I’ in Figure 6) would undergo the evaporation process each scan, cluster and re-deposit back mostly on the tips of the CNT conic bundles (Figure 1).

The gradual step-by-step ablation leads to coalescence and increase in the diameter of the nanoparticles formed during the first FSL scans. At a certain diameter of nanospheres, due to Gaussian distribution of laser intensity, the incident energy might be not enough to evaporate the nanospheres completely and they undergo melting instead. Being in a liquid state, they wet the surrounding CNTs. Once the FSL irradiation is stopped, they freeze together forming the observed Fe phase nanosphere/conical CNT bundle nanostructures (Fe/CNT nanostructures), while the graphitic shells (if any) of a very complicated structure (Figure 3a) are being extruded during their cooling (Figure 6 (4)).

This scenario of the Fe/CNT nanostructure shape formation is the most realistic because it clearly explains why in most cases Fe nanospheres are located directly on the tips of the CNT cones. It is worth mentioning, however, that at the beginning, the electrostatic forces between CNTs are responsible for the formation of the CNT cones structure because sometimes the nanospheres are too small to be able to link the nearby CNTs just by wetting, which was observed in other works also [25].

The described mechanism is the most realistic due to another reason since there is no clear periodicity of the shape of the Fe/CNT nanostructures like, for example, in the case of ‘black silicon’ where the cone formation is governed by the initial ripple creation with the wavelength close to the central wavelength of the incident laser [7].

## Conclusions

In the present work, we investigated for the first time the interaction of FSL irradiation with the arrays of vertically aligned carbon nanotubes intercalated with the ferromagnetic (Fe phase) nanoparticles. The presence of metal nanoparticles in CNT array plays the main role in the energy absorption by the array. As a result of such interaction, a novel composite nanostructured material was obtained. This nanomaterial consists of tiny Fe phase nanospheres attached to the tips of the CNT bundles of conical shape. We designated this material as Fe phase nanosphere/conical CNT bundle nanostructures. The mechanism of such nanostructure formation was proposed. The importance of the present investigation is defined by the possible applications of the obtained results. The arrays of CNTs with the intercalated ferromagnetic nanoparticles, i.e., MFCNTs, may be considered as an ideal medium for different magnetic applications. The FSL irradiation may become an instrument for the machining of the mentioned devices based on the arrays of MFCNTs. Moreover, one could expect that the obtained nanostructures would possess new optical properties which would find applications in photovoltaics and plasmonics.

## Abbreviations

CNT: Carbon nanotube; CVD: Chemical vapor deposition; EDX spectroscopy: Energy-dispersive X-ray spectroscopy; Fe/CNT nanostructures: Fe phase nanosphere/conical CNT bundle nanostructures; FSL: Femtosecond laser; SEM: Scanning electron microscopy; TEM: Transmission electron microscopy; XRD: X-ray diffraction.

## Competing interests

The authors declare that they have no competing interests.

## Authors’ contributions

VL coordinated the study, analyzed the data, and contributed to the manuscript preparation. AP synthesized the CNT arrays, performed structural analyses of the samples, analyzed the experimental results, and contributed to the manuscript preparation. SB carried out the femtosecond laser irradiation of the CNT arrays and analyzed the data. SF performed EDX study of the irradiated CNTs. BS and BKT analyzed the data and contributed to the manuscript preparation. YS and AB carried out TEM and analyzed the data. All authors read and approved the final manuscript.
